# Dynamics of cinacalcet use and biochemical control in hemodialysis patients: a retrospective New-user cohort design

**DOI:** 10.1186/s12882-015-0174-6

**Published:** 2015-10-29

**Authors:** B. Diane Reams, Paul J. Dluzniewski, Thy P. Do, Susan V. Yue, Brian D. Bradbury, Abhijit V. Kshirsagar, M. Alan Brookhart

**Affiliations:** Cecil G. Sheps Center for Health Services Research, University of North Carolina, Chapel Hill, NC USA; Amgen, Inc, Thousand Oaks, CA USA; University of North Carolina Kidney Center, UNC School of Medicine, Chapel Hill, NC USA; Department of Epidemiology, UNC Gillings School of Global Public Health, UNC Chapel Hill, Chapel Hill, NC USA

**Keywords:** Cinacalcet, Secondary hyperparathyroidism, Hemodialysis

## Abstract

**Background:**

Cinacalcet is used to treat secondary hyperparathyroidism among hemodialysis patients. Large-scale epidemiologic studies describing patterns of cinacalcet use, effects on parathyroid hormone (PTH), calcium, and phosphorous levels, and predictors of discontinuation have not been previously reported.

**Methods:**

This retrospective cohort study used a clinical database of a large U.S. dialysis provider (2007–2010) merged with administrative data from the United States Renal Data System. Among new users of cinacalcet with Medicare coverage, trends in PTH, calcium, and phosphorus were measured in 30-day intervals following cinacalcet initiation.

**Results:**

Seventeen thousand seven hundred sixty-three eligible initiators contributed 111,047 30-day follow-up intervals. Of these, 56 % discontinued cinacalcet by month 4. Of those discontinuing, 76.3 % reinitiated. Mean values of PTH, calcium, and phosphorus decreased to recommended levels within 4 months following initiation. Proximal PTH levels <150 pg/mL were associated with discontinuation: HR = 1.23 (95 % CI: 1.12, 1.36), whereas low calcium (<7.5 mg/dL) was suggestive of an association, HR = 1.09 (95 % CI 0.91, 1.32). Being in the Part D gap period increased discontinuation risk: HR = 1.09 (95 % CI: 1.03, 1.16). Low-income subsidy status decreased discontinuation risk: HR = 0.77 (95 % CI 0.69, 0.86). Predictors of reinitiation included low-income subsidy, HR = 1.32 (95 % CI 1.22, 1.43); higher albumin level, HR = 1.23 (95 % CI 1.10, 1.36) and higher calcium level, HR = 1.26 (95 % CI 1.19, 1.33).

**Conclusions:**

Substantial and expected declines in laboratory values occurred following cinacalcet initiation. Early discontinuation and reinitiation of cinacalcet were common and may have occurred for clinical and economic reasons.

**Electronic supplementary material:**

The online version of this article (doi:10.1186/s12882-015-0174-6) contains supplementary material, which is available to authorized users.

## Background

Secondary hyperparathyroidism (SHPT) among patients with chronic kidney disease (CKD) results from decreased active vitamin D and is manifested by low serum calcium and elevated levels of phosphorus and parathyroid hormone (PTH) [1, 2]. Treatment options to control CKD-MBD parameters include modulation of calcium and phosphorous balance through dietary intake and dialysis, active vitamin D compounds, and phosphate binders [3]. The Kidney Disease: Improving Global Outcomes (KDIGO) guideline recommendations for the optimal range of PTH is two to nine times the assay's upper limit of normal reference range, with levels not to exceed approximately 600 pg/mL. Practitioners are also recommended to target the assay reference range for calcium and phosphorus [4].

Another therapeutic option is cinacalcet (Sensipar®/Mimpara®, Amgen Inc., Thousand Oaks, CA), a calcimimetic agent that directly lowers PTH, and subsequently, calcium and phosphorus [5, 6]. However, post-marketing studies in real world settings have found adherence to cinacalcet to be sporadic, which may limit its effectiveness [7–9]. Identifying factors associated with discontinuation may help healthcare providers better understand and prevent non-adherence to therapy. Previous studies examining adherence to cinacalcet have been limited by size, the inability to identify time-varying covariates, and inaccuracies in identifying therapy start and stop dates [7, 10–12].

Using detailed clinical, laboratory, and healthcare utilization data from a large cohort of patients receiving hemodialysis, we sought to describe the experience of patients initiating cinacalcet, including the trajectory of biochemical parameters following initiation, as well as factors predicting discontinuation and reinitiation of therapy.

## Methods

### Data sources

Our study used 4 years of data (January 1, 2007 through December 31, 2010) from the United States Renal Data System (USRDS) linked to data from a large dialysis provider. The dialysis provider owns and manages over 1,500 outpatient dialysis facilities located throughout the U.S. in urban, rural, and suburban areas. Their clinical database captures detailed clinical, laboratory, and treatment data on patients receiving care at all of their dialysis units. Data are collected using standardized clinical protocols and electronic record systems. The USRDS is a national data system, funded by the National Institute of Diabetes and Digestive and Kidney Diseases, which collects, analyzes, and distributes information about the treatment of end-stage renal disease (ESRD). The USRDS data include data from the Medical Evidence Report Form, Medicare Enrollment database, ESRD Death Notification Form, as well as standard analytic files that contain final action claims for Medicare Parts A, B, and D services [13]. Medicare Parts A through D are government provided insurance programs for the elderly, disabled, and those with certain medical conditions. Permission to use USRDS data was obtained from The National Institute of Diabetes and Digestive Kidney Diseases. These data were used to obtain information on demographic and comorbid characteristics and health care utilization, such as hospitalizations and outpatient care.

### Study design

Our study used a retrospective new-user cohort design [14]. Patients were eligible to be included in our study if they were 18 years and older, had one year of continuous Medicare Parts A, B, and D coverage during the study period, and had in-center hemodialysis at the large dialysis provider’s facility. During a 6-month baseline period, we identified potential confounders and effect modifiers. Among all eligible patients, new cinacalcet users were defined as patients who filled at least one 30-day cinacalcet prescription without use in the prior 6 months (baseline period) based on USRDS Medicare Part D claims files. New users were also required to have received hemodialysis for at least 9 months and had at least 9 dialysis sessions during the last month of the baseline period. Patients receiving a parathyroidectomy during the baseline period or 1st 30-day fill of cinacalcet were excluded. The cohort construction is outlined in Additional file 1: Figure S1.

The start of follow-up was on day 31 following an initial 30-day fill of any dose of cinacalcet. At the end of each 30-day interval, the patient’s treatment status was determined. Patients were defined as discontinuing cinacalcet if there was greater than a 30-day gap from their last pill day, calculated from the days supply dispensed. Patients were then followed from discontinuation until first reinitiation of cinacalcet. Because we did not know the exact date of discontinuation or reinitiation in a given interval, the 30-day interval prior to the discontinuation or reinitiation interval was used for the identification of time-varying covariates including laboratory values, cardiovascular and renal risk factors, and medications. The most recent laboratory or covariate information in the 30-day interval prior to discontinuation or reinitiation was documented. If laboratory information was not available in the 30-day interval, the most recent value in the prior 90 days was documented. Study schemata are provided in Additional file 1: Figures S2 and S3. In addition, changes in quintile distributions of PTH, calcium, and phosphorus were examined over time in order to assess how increases and decreases between quintiles predict discontinuation and reinitiation. A sensitivity analysis was performed to determine if reinitiation results were modified when biochemical results from 5, 7, and 14 days prior to the date of the laboratory value most proximal to reinitiation were used to predict reinitiation. This lag time could account for any delays between physician recognition of a laboratory abnormality and a decision to reinitiate cinacalcet.

### Study variables

#### Outcomes

Our outcomes of interest were PTH, calcium, and phosphorus serum laboratory values measured monthly following cinacalcet initiation and discontinuation, as well as those leading up to reinitiation of cinacalcet.

#### Covariates

Descriptions and definitions of baseline and time-varying covariates are provided in Additional file 1: Table S1 and Table S2. We identified from Medicare and linked clinical data demographic, laboratory, and clinical variables, as well as comorbidities. Covariates included demographic characteristics (e.g., age, sex, race, Medicaid eligibility, census region, year), clinical characteristics (e.g., cause of ESRD, time on dialysis, body mass index, type of vascular access, number of hospital days), baseline laboratory variables (e.g., PTH, calcium, phosphorus), time-varying laboratory variables divided into categories consistent with recognized normal and abnormal values, and several time-varying comorbidity measures. International Classification of Diseases Ninth Revision codes, Current Procedural Terminology codes, and Healthcare Common Procedure Coding System codes were used to identify comorbidities and procedures.

### Statistical analysis

To describe the treatment groups, we report means and frequencies of all covariates within each treatment group and subgroups of interest. Trends in means for each lab value were plotted for the months after initiation and discontinuation. These plots were smoothed and 95 % confidence intervals computed using smoothing splines. For each 30-day interval, we fit a logistic model and estimated the probability of discontinuation or reinitiation given the most recent time-varying covariates in the 90 days prior to the 30-day interval in which discontinuation or reinitiation was thought to have occurred. Although each patient could contribute multiple 30-day intervals to the analytic dataset, we did not adjust for repeated measures since each patient, per our study design, can only discontinue once. Patients were censored administratively on December 31, 2010, and for loss to follow-up, transplant, discontinuation of hemodialysis, death, parathyroidectomy, or loss of Medicare Parts A, B, or D coverage. We also described trends in biochemical parameters following treatment discontinuation and leading up to reinitiation across a range of *a priori* identified subgroups, including those defined by age, time on dialysis, and race.

This research was determined to be exempt from review by the UNC Institutional Review Board. All statistical analyses were conducted using the R Statistical Software version 3.3 [15] and SAS software, Version 9.3, SAS Institute Inc., Cary, NC, USA.

## Results

We identified 17,763 patients who met our study entry requirements and contributed 111,047 30-day follow-up intervals. Table 1 presents patient characteristics of the primary cohort stratified by gender. All covariates considered can be found in Additional file 1: Table S3. At cinacalcet initiation, the average age was 56.7 years (standard deviation (SD) 14.5 years) and the average time on dialysis was 4.5 years (SD 4.3 years), 49.3 % of the cohort was female, and 53.8 % were African American. Several baseline financial factors were also identified. A history of receiving Medicaid benefits or having low-income subsidy was identified in 68.7 and 83.9 %, respectively. Mean PTH calcium, and phosphorus at initiation were 642 pg/mL (SD 519 pg/mL), 9.4 mg/dL (SD 0.7 mg/dL), and 5.9 mg/dL (SD 1.7 mg/dL), respectively.

**Table 1:** Baseline characteristics overall and by gender

Characteristic^a^	Total	Female	Male
Demographics			
Patients, *N*	17,763	8,764	8,999
Age, mean (SD), years^b^	56.7 (14.5)	59.1 (14.8)	54.4 (13.9)
Time on dialysis, mean (SD), years^b^	4.5 (4.3)	4.4 (4.1)	4.7 (4.4)
Race, *N* (%)			
White	7,242 (40.8)	3,436 (39.2)	3,806 (42.3)
African American	9,555 (53.8)	4,856 (55.4)	4,699 (52.2)
Other Race	966 (5.4)	472 (5.4)	494 (5.5)
Cause of ESRD, *N* (%)			
Diabetes	7,629 (42.9)	4,233 (48.3)	3,396 (37.7)
Hypertension	5,612 (31.6)	2,458 (28.0)	3,154 (35.0)
Glomerulonephritis	2,236 (12.6)	1,067 (12.2)	1,169 (13.0)
Other	2,286 (12.9)	1,006 (11.5)	1,280 (14.2)
Body Mass Index, mean (SD), kg/m^2^	28.0 (7.3)	28.7 (7.8)	27.4 (6.7)
Financial Considerations			
Medicaid, *N* (%)	12,206 (68.7)	6,351 (72.5)	5,855 (65.1)
Low-income subsidy, *N* (%)	14,906 (83.9)	7,515 (85.7)	7,391 (82.1)
Concomitant medications, *N* (%)^c^	4.7 (3.6)	5.1 (3.7)	4.4 (3.5)
Biochemical Values			
Albumin, mean (SD), g/dL^d^	3.9 (0.4)	3.8 (0.4)	4.0 (0.4)
Calcium, mean (SD), mg/dL^d^	9.4 (0.7)	9.4 (0.7)	9.4 (0.7)
Phosphorus, mean (SD), mg/dL^d^	5.9 (1.7)	5.8 (1.7)	6.0 (1.7)
Parathyroid hormone, mean (SD), pg/mL^d^	642 (519)	640 (519)	644 (520)
Comorbidities			
Congestive heart failure, *N* (%)	4,823 (27.2)	2,592 (29.6)	2,231 (24.8)
Coronary artery disease/atherosclerosis, *N* (%)	4,703 (26.5)	2,434 (27.8)	2,269 (25.2)
Cerebrovascular disease, *N* (%)	1,891 (10.6)	1,087 (12.4)	804 (8.9)
Hypertension, *N* (%)	12,393 (69.8)	6,481 (74.0)	5,912 (65.7)
Peripheral vascular disease, *N* (%)	2,352 (13.2)	1,208 (13.8)	1,144 (12.7)
Hyperlipidemia, *N* (%)	4,658 (26.2)	2,519 (28.7)	2,139 (23.8)
Chronic obstructive pulmonary disease or asthma, *N* (%)	2,727 (15.4)	1,530 (17.5)	1,197 (13.3)
Diabetes, *N* (%)	10,052 (56.6)	5,491 (62.7)	4,561 (50.7)
Dialysis Care			
Phosphorus binder drug, *N* (%)^e^	14,135 (79.6)	7,037 (80.3)	7,098 (78.9)
Catheter access, *N* (%)	3,351 (18.9)	1,994 (22.8)	1,357 (15.1)
Mean intravenous vitamin D dosage, micrograms (SD)^f^	12.5 (10.3)	11.9 (9.9)	13.1 (10.7)

*Note:* Conversion factors for units: Calcium in mg/dL to mmol/L, x0.2495; phosphorus in mg/dL to mmol/L, x0.3229

^a^Characteristics were identified using information from Medicare Part A or B claims. A characteristic was considered present if at least one inpatient, home health, or skilled nursing facility claim, or at least two outpatient or physician/supplier claims separated by at least 7 days, were identified during the 6-month baseline period. Additional information concerning covariates can be found in Additional file 1: Table S3

^b^Age and time on dialysis are at the time of cinacalcet initiation

^c^Concomitant medications are the number of concomitant medications at the time of cinacalcet initiation

^d^Laboratory values were those most proximal to the index date during the baseline period

^e^Phosphate binders included in the analysis: Sevelamer hydrochloride, sevelamer carbonate, lanthanum carbonate, and calcium acetate

^f^Mean intravenous vitamin D dose per person in the last month of the baseline period. Paricalcitol and doxercalciferol doses were converted to calcitriol-equivalent doses according to the following conversion ratios: 4.6: 1 for paricalcitol: calcitriol and 3.1: 1 for doxercalciferol: calcitriol

**Fig. 1:**
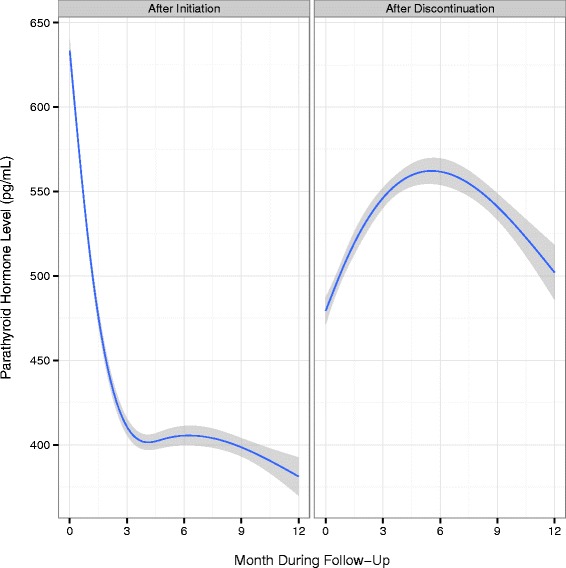
Mean PTH levels and 95 % confidence intervals by month following cinacalcet initiation and discontinuation

**Fig. 2:**
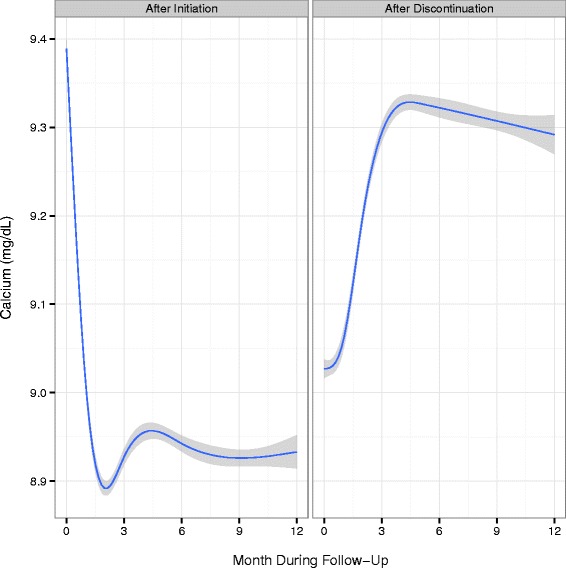
Mean calcium levels and 95 % confidence intervals by month following cinacalcet initiation and discontinuation. *Note:* Conversion factors for units: Calcium in mg/dL to mmol/L, x0.2495

**Fig. 3:**
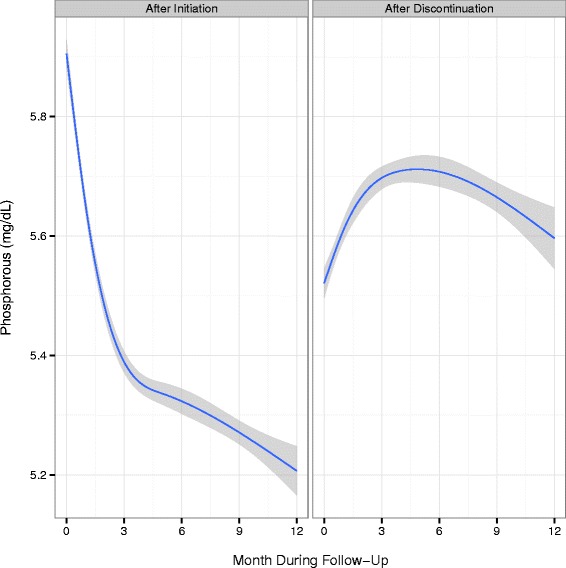
Mean phosphorus levels and 95 % confidence intervals by month following cinacalcet initiation and discontinuation. *Note:* Conversion factors for units: phosphorus in mg/dL to mmol/L, x0.3229

Trends in the mean serum lab values of PTH, calcium, and phosphorus for the first 12 months following cinacalcet initiation, as well as after discontinuation, are shown in Figs. 1, 2 and 3. Following cinacalcet initiation, the levels of the three lab values fell and stabilized at approximately month 4. Mean PTH levels decreased from a baseline level of 642 pg/mL (SD 519 pg/mL) to levels between 375 and 400 pg/mL in months 4 through 12. Mean calcium levels decreased from a baseline level of 9.4 mg/dL (SD 0.7 mg/dL) to approximately 8.9 mg/dL during the 1st month and remained at levels slightly higher than 8.9 mg/dL between months 4 through 12. Of the 111,047 30-day intervals examined, 17,980 intervals (16.7 %) had a PTH level less than 150 pg/mL and 1,934 (1.8 %) had a calcium level less than 7.5 mg/dL, Additional file 1: Table S4. Phosphorus levels decreased from a baseline mean level of 5.9 mg/dL (SD 1.7 mg/dL) to between 5.2 and 5.4 mg/dL in months 4 through 12. Following discontinuation, mean levels of PTH, calcium, and phosphorus increased and then stabilized at higher mean levels than those achieved while on therapy.

The probability of discontinuation by month 4 was 56 % and by month 12 was 73 %. Of those who discontinued (*N* = 12,521), 76.3 % (*N* = 9,558) reinitiated cinacalcet. The mean time to reinitiation was 4.0 months. Predictors of cinacalcet discontinuation and reinitiation are presented in Table 2. All covariates considered can be found in Additional file 1: Table S5. Baseline PTH, calcium, and phosphorus serum levels were not associated with discontinuation; however, levels most proximal to discontinuation were. Low proximal levels of PTH (<150 pg/mL) were associated with discontinuation, HR 1.23 (95 % CI 1.12, 1.36). There was a slight association between calcium (<7.5 mg/dL) and discontinuation, HR 1.09 (95 % CI 0.91, 1.32). Increasing levels of PTH and calcium over time, based on changes in quintile distributions, were also associated with discontinuation, HR 1.15 (95 % CI 1.07, 1.23) and HR 1.24 (95 % CI 1.16, 1.32), respectively. The dose of cinacalcet most proximal to discontinuation also predicted discontinuation in this population. Compared to 30 mg, a recent dose of 60 mg of cinacalcet slightly predicted discontinuation, HR 1.07 (95 % CI 1.00, 1.15), while a recent dose of 90 mg compared to 30 mg had a stronger association, HR 1.15 (95 % CI 1.03, 1.29). Other factors associated with discontinuation included increasing copay, in the follow-up period, HR 1.04 (95 % CI 1.02, 1.07); time spent in the hospital, HR 2.02 (95 % CI 1.84, 2.22); being in the Medicare Part D gap period, HR 1.09 (95 % CI 1.03, 1.16); and a diagnosis of stroke during follow-up, HR 1.30 (95 % CI 1.06, 1.60). Nausea, vomiting, and diarrhea were not common in our study given the limitation of ICD-9 diagnosis codes to identify these outcomes, *N* = 1,308 30-day intervals (1.2 %) and were only slightly predictive of discontinuation, HR 1.09 (95 % CI 0.91, 1.32). Proximal PTH values in all categories examined did not predict reinitiation of cinacalcet. Both increasing and decreasing levels of PTH over time, based on changes in quintile distributions, were both associated with reinitiation of PTH, HR 1.08 (95 % CI 1.03, 1.14) and HR 1.12 (95 % CI 1.06, 1.19), respectively. Proximal calcium levels both lower and higher were predictive of cinacalcet reinitiation; however, the results of the sensitivity analysis, which used the calcium value 14 days prior to the date of the laboratory value most proximal to reinitiation, showed that only higher calcium levels were associated with reinitiation, HR 1.26 (95 % CI 1.19, 1.33), Additional file 1: Table S6. All other sensitivity analysis results were not greatly changed when prior laboratory values were used to predict reinitiation. Other predictors of reinitiation included low-income subsidy, HR = 1.32 (95 % CI 1.22, 1.43), African American race, HR = 1.08 (95 % CI 1.03, 1.13), and higher albumin level, HR = 1.23 (95 % CI 1.10, 1.36).

**Table 2:** Predictors of discontinuation and reinitiation

Characteristic^a^	Discontinuation (HR, 95 % CI)	Reinitiation (HR, 95 % CI)
Number of time intervals for analysis, *N* (%)	100,706 (90.7 %)	78,789 (96.0 %)
Demographics		
Age, years, reference 46-55		
≤45	0.92 (0.85, 1.00)	0.95 (0.90, 1.02)
56-65	1.05 (0.97, 1.13)	0.98 (0.92, 1.04)
66-75	1.05 (0.97, 1.15)	0.90 (0.85, 0.97)
≥75	0.98 (0.88, 1.09)	0.95 (0.87, 1.04)
Time on dialysis, years, reference <1		
1-3	1.15 (1.01, 1.30)	1.00 (0.91, 1.11)
≥4	1.14 (1.00, 1.30)	1.03 (0.92, 1.14)
Female	1.07 (1.01, 1.13)	1.00 (0.96, 1.05)
African American	1.04 (0.98, 1.10)	1.08 (1.03, 1.13)
Cause of ESRD, reference diabetes mellitus		
Hypertension	1.02 (0.94, 1.10)	1.04 (0.98, 1.11)
Glomerulonephritis	1.01 (0.90, 1.12)	1.08 (0.99, 1.17)
Other	0.96 (0.87, 1.06)	1.03 (0.95, 1.12)
Body mass index, kg/m^2^, reference normal		
Underweight	1.07 (0.93, 1.23)	0.98 (0.87, 1.10)
Overweight	1.03 (0.96, 1.10)	1.03 (0.98, 1.09)
Obese	0.98 (0.91, 1.04)	1.11 (1.05, 1.17)
Financial considerations		
Medicaid	1.03 (0.96, 1.11)	0.96 (0.91, 1.02)
Low-income subsidy	0.76 (0.68, 0.85)	1.32 (1.22, 1.43)
Concomitant medications in baseline period^b^	0.98 (0.97, 0.99)	1.00 (0.99, 1.01)
Concomitant medications in follow-up period^b^	0.96 (0.95, 0.97)	0.98 (0.97, 0.99)
Copay in follow-up period^c^	1.04 (1.01, 1.06)	1.04 (1.02, 1.06)
Last benefit phase in follow-up, reference: covered^d^		
Entering the gap period	1.19 (1.00, 1.41)	1.01 (0.85, 1.21)
Exiting or going through gap period	0.98 (0.77, 1.23)	1.03 (0.81, 1.32)
In the gap period	1.10 (1.04, 1.16)	1.01 (0.96, 1.06)
Biochemical values		
Albumin in baseline period, reference: <3.3 g/dL		
3.3-3.9 g/dL	1.11 (0.97, 1.28)	1.13 (1.00, 1.27)
>3.9 g/dL	1.05 (0.91, 1.21)	1.09 (0.96, 1.23)
Albumin in follow-up period, reference: <3.3 g/dL		
3.3-3.9 g/dL	0.85 (0.76, 0.95)	1.13 (1.02, 1.25)
>3.9 g/dL	0.78 (0.69, 0.88)	1.23 (1.10, 1.36)
Phosphorus in baseline period, mg/dL	1.02 (1.00, 1.04)	0.98 (0.96, 0.99)
Phosphorus in follow-up period, mg/dL	1.02 (1.00, 1.04)	0.99 (0.98, 1.01)
Parathyroid hormone in baseline period, pg/mL^e^	1.00 (0.99, 1.01)	1.00 (0.99, 1.00)
Parathyroid hormone in follow-up period, reference: >600 pg/mL		
<150 pg/mL	1.24 (1.12, 1.37)	0.70 (0.64, 0.76)
150-300 pg/mL	0.91 (0.83, 0.99)	0.71 (0.66, 0.75)
301-600 pg/mL	0.89 (0.82, 0.97)	0.85 (0.80, 0.90)
Parathyroid hormone in follow-up period, change in quintiles, reference: no change^f^		
Increase	1.15 (1.07, 1.23)	1.08 (1.03, 1.14)
Decrease	0.90 (0.84, 0.97)	1.12 (1.06, 1.19)
Calcium in baseline period, mg/dL	0.95 (0.91, 0.99)	1.16 (1.12, 1.20)
Calcium in follow-up period, reference: >8.7 mg/dL^g^		
<7.5 mg/dL	1.07 (0.89, 1.29)	1.12 (0.91, 1.39)
7.5-8.7 mg/dL	0.85 (0.80, 0.91)	1.26 (1.19, 1.33)
Calcium in follow-up period, change in quintiles, reference: no change^f^		
Increase	1.24 (1.16, 1.32)	1.07 (1.02, 1.13)
Decrease	0.94 (0.88, 1.00)	1.04 (0.99, 1.10)
Comorbidities		
Congestive heart failure in baseline period	1.06 (0.99, 1.13)	0.96 (0.91, 1.01)
Congestive heart failure in follow-up period	1.01 (0.90, 1.14)	1.11 (0.94, 1.31)
Coronary artery disease/atherosclerosis in baseline period	1.01 (0.95, 1.09)	0.93 (0.88, 0.99)
Cerebrovascular disease in baseline period	0.94 (0.86, 1.02)	1.00 (0.93, 1.07)
Stroke in follow-up period	1.30 (1.05, 1.60)	0.82 (0.55, 1.20)
Hypertension in baseline period	1.12 (1.05, 1.19)	1.03 (0.98, 1.08)
Peripheral vascular disease in baseline period	1.02 (0.94, 1.11)	1.11 (1.03, 1.19)
Peripheral vascular disease in follow-up period	0.99 (0.85, 1.16)	0.91 (0.76, 1.09)
Hyperlipidemia in baseline period	1.01 (0.95, 1.07)	1.06 (1.01, 1.12)
Chronic obstructive pulmonary disease and asthma in baseline period	1.00 (0.93, 1.08)	1.00 (0.94, 1.06)
Diabetes in baseline period	1.05 (0.98, 1.14)	0.98 (0.92, 1.04)
Nausea, vomiting, diarrhea in follow-up period	1.09 (0.91, 1.32)	1.05 (0.85, 1.31)
Seizure in follow-up period	1.18 (0.93, 1.49)	1.11 (0.82, 1.50)
Dialysis care		
Intravenous vitamin D in baseline period^h^	1.01 (0.98, 1.05)	0.98 (0.96, 1.00)
Intravenous vitamin D in follow-up period^h^	0.94 (0.91, 0.97)	1.02 (0.99, 1.04)
Phosphorus binder drug in baseline period^i^	1.02 (0.95, 1.10)	1.12 (1.06, 1.18)
Phosphorus binder drug in follow-up period^i^	0.77 (0.73, 0.82)	1.03 (0.98, 1.08)
Catheter access in baseline period	0.97 (0.89, 1.07)	1.07 (0.99, 1.15)
Catheter access in follow-up period	1.10 (1.00, 1.21)	0.83 (0.77, 0.90)
Most recent dose of cinacalcet, reference: 30 mg		
60 mg	1.07 (1.00, 1.15)	n/a
90 mg	1.15 (1.03, 1.29)	n/a
Days in the hospital in follow-up period, reference: 0 days		
1-4 days	2.02 (1.84, 2.22)	0.85 (0.75, 0.96)
≥5 days	1.90 (1.73, 2.08)	0.79 (0.69, 0.89)

*Note:* Conversion factors for units: Calcium in mg/dL to mmol/L, x0.2495; phosphorus in mg/dL to mmol/L, x0.3229

^a^Baseline characteristics were identified using information from Medicare Part A or B claims. A characteristic was considered present if at least one inpatient, home health, or skilled nursing facility claim, or at least two outpatient or physician/supplier claims separated by at least 7 days, were identified during the 6-month baseline period. Additional information concerning baseline characteristics can be found in Additional file 1: Table S4. Time-varying (follow-up) characteristics were evaluated at 30-day intervals following the start of follow-up. Additional information concerning time-varying (follow-up) characteristics can be found in Additional file 1: Table S2

^b^Concomitant medications are the number of concomitant medications at the time of cinacalcet discontinuation or reinitiation

^c^Changes in co-pay were based on increments of $100. The last co-pay prior to discontinuation was used to predict cinacalcet reinitiation

^d^Benefit phase reflects the status of Medicare Part D coverage at the time of the fill of cinacalcet

^e^Changes in parathyroid hormone level were based on increments of 100 pg/mL

^f^Distributions of parathyroid hormone and calcium were examined across all of follow-up and quintiles were based on these distributions. Increase indicates an increase to another quintile and trend of increasing laboratory levels. Decrease indicates a decrease to another quintile and a trend of decreasing laboratory levels

^g^Results presented for prediction of reinitiation associated with follow-up calcium levels are those from the sensitivity analysis utilizing a lag time of 14 days. The calcium level recorded 14 days prior to the date of the laboratory value most proximal to discontinuation was used to predict reinitiation. All other results were not significantly changed when lag times were considered

^h^Mean intravenous vitamin D dose was assessed in the last month of the baseline period. Changes in intravenous vitamin D dose were based in increments of 10mcg. Paricalcitol and doxercalciferol doses were converted to calcitriol-equivalent doses according to the following conversion ratios: 4.6: 1 for paricalcitol: calcitriol and 3.1: 1 for doxercalciferol: calcitriol

^i^Phosphate binders included in the analysis: Sevelamer hydrochloride, sevelamer carbonate, lanthanum carbonate, and calcium acetate

## Discussion

In a cohort of contemporary hemodialysis patients, we observed the effectiveness of cinacalcet in lowering levels of PTH, calcium, and phosphorus, and determined predictors of early cinacalcet discontinuation and subsequent re-initiation. The reductions in these lab values are observed soon after initiation of cinacalcet and mean levels of PTH, calcium, and phosphorus appear to be sustained within recommended target ranges. Yet, discontinuation occurs frequently and relatively soon after initiation of cinacalcet treatment, on average within 4 months following initiation, Additional file 1: Table S7. Furthermore, discontinuation in this population resulted in the loss of biochemical control. Several variables predicted discontinuation. Some of these variables were biochemical, including declining PTH and calcium levels, and others were related to drug prescription drug coverage and novel findings associated with changes in health status. Variables predicting reinitiation were generally similar to those predicting discontinuation.

Average PTH, calcium, and phosphorus levels following cinacalcet initiation in our study were consistent with the 2009 KDIGO guideline update, despite a portion of the follow-up period occurring prior to the release of the guidelines [4]. These guidelines suggest a PTH level in the range of approximately 2 to 9 times the upper reference limit for the assay, which corresponds to approximately 130 to 600 pg/mL, taking into account variability in commercial assays. In our study, average serum calcium levels were maintained in the reference range, in which the recommended upper level of the range is 10.0 mg/dL to 10.5 mg/dL, depending on the assay used. Average phosphorus levels in our study decreased toward the reference range, which is 2.4 to 4.1 mg/dL, differing slightly among assays. For patients who remained on cinacalcet, biochemical control persisted for up to one year of follow-up. Our results are also similar to those from a recent retrospective cohort study that showed decreases in all three biochemistries that were sustained for 1-year following cinacalcet initiation, although we did not assess dose-titration and its impact on control [7]. Following discontinuation of cinacalcet in the current study, biochemical values increased quickly then leveled off or decreased slightly, Figs. 1, 2 and 3. The slight decreases in biochemical levels are likely due to a selection effect that would result from patients with higher levels restarting treatment and thus being censored from the analysis. Similar to a prior study [16], vitamin D doses in our population decreased following cinacalcet initiation and increased following cinacalcet discontinuation, Additional file 1: Figure S4.

Despite evidence of improved control of SHPT-related biochemical parameters, discontinuation of cinacalcet was common, with 56 % discontinuing by month 4 and 73 % discontinuing by month 12. This rate of discontinuation is greater than what has been reported in previous cinacalcet studies. Although we cannot know the precise cause of discontinuation, our study is the first to include a large sample size and verify use through Medicare Part D prescription claims, which overcomes many of the possible inaccuracies in start and stop dates [7, 10–12]. Prior studies of medication adherence in the general and CKD populations have found that >50 % of patients demonstrate poor adherence to chronic medications [17]. While poor patient adherence is possible in our population, reinitiation in our population was common and on average occurred 4 months after the first discontinuation. These findings suggest that discontinuation is due to several factors and likely to encompass both patient-initiated (non-adherence) and physician-initiated, medically driven (biochemical value influences) factors.

Our study identified possible predictors that may illuminate why certain patients are discontinued from cinacalcet. While the prescribing information for cinacalcet indicates that administration should be withheld if serum calcium falls below 7.5 mg/dL, only a small proportion of patients experienced hypocalcemia and the modeling revealed it as a weak predictor of discontinuation. PTH levels falling below 150 pg/mL was more common and was predictive of discontinuation, consistent with prescribing information. Higher doses of cinacalcet at the time of discontinuation also predicted discontinuation, which may further illustrate the role of low or at goal laboratory values in the decision to discontinue cinacalcet. Other possible cinacalcet side effects, nausea, vomiting, and diarrhea [18], were also not common in our study, but did have a weak association with discontinuation. However, because nausea and vomiting are likely to be under reported, measuring this well-recognized side effect is difficult and definitive conclusions from the observed results cannot be drawn for this variable.

We also identified factors related to medication cost that were predictive of discontinuation and reinitiation, suggesting that financial issues could play a role in patients’ decisions to discontinue and reinitiate treatment. Patients identified as ever having low-income subsidy status, indicating a lower-out-of pocket cost for cinacalcet, were less likely to discontinue and more likely to reinitiate therapy following discontinuation. Entering the Medicare Part D gap period or being in the Medicare Part D gap period, when out-of-pocket costs are significant, increased the risk of discontinuation. Increasing copay during follow-up also slightly increased the risk of discontinuation. Our results are similar to the findings of a recent study of adherence and persistence of oral medications, defined as ≥ 80 % medication possession ratio and duration of use, among Medicare Part D beneficiaries receiving dialysis [19]. The investigators found patients not receiving the low-income subsidy and patients entering into the coverage gap were more likely to be non-adherent and had less persistent use of cinacalcet [19]. The investigators found similar relationships with other oral medications indicating that the impact of economic burden in this population is not specific to cinacalcet. Therapy regimens for patients receiving dialysis are burdensome, and patients are prescribed on average 10–12 tablets per day, all likely leading to financial burden [10, 20, 21].

It has been recommended that therapeutic decisions in CKD patients be based on trends, rather than single laboratory values [4]. Therefore, in addition to examining the laboratory values most proximal to the time of discontinuation, we also examined the changes in lab values leading up to discontinuation. Our most striking finding was that trends of increasing levels of PTH and calcium over time were associated with discontinuation. This finding could be an early signal of non-adherence. As patients become non-adherent to cinacalcet due to side effects, financial issues, or medication complexity, rising levels of PTH and calcium could be apparent and a signal to clinicians to consider that factors other than medication ineffectiveness may be an issue. Identifying ways to decrease medication complexity, financial burdens, and side effects could enable patients to persist longer on cinacalcet therapy.

Several limitations of our study must be noted. First, medication use information was obtained from pharmacy claims, which are an imperfect measure of actual medication consumed. It is possible that patients were obtaining medications outside of their Medicare Part D benefit [22, 23]. We also cannot precisely determine the day when patients stops or reinitiates treatment. However, we adopted a design to minimize the likelihood that predictors would be assessed following discontinuation or reinitiation events, thus avoiding the problem of predictors being consequences of discontinuation or reinitiation, rather than causes. In addition, we performed a sensitivity analysis to determine if a delay in physician recognition and response to a laboratory value could affect reinitiation results. Adding a lag in the dates of the biochemical values affected calcium reinitiation results, a biochemical test drawn frequently, but not PTH results, a biochemical test drawn less frequently. In future studies using claims data, consideration of the time frame of when frequent biochemical values are drawn and when a physician would realistically identify and act on a biochemical value should be considered. Finally, many of our variables were assessed using ICD-9 codes associated with health care encounters. Many of these definitions, such as those for acute myocardial infarction [24], are known to have very high sensitivity and specificity, but others, such as those for nausea, are likely to be much less sensitive.

## Conclusions

The results of our study indicate that on average biochemical control of PTH, calcium, and phosphorus following cinacalcet initiation were consistent with recommendations in the 2009 KDIGO guideline update. Yet, early discontinuation of cinacalcet was frequent and resulted in the loss of control of PTH, calcium, and phosphorus. Both economic and clinical factors contribute to cinacalcet discontinuation, as well as reinitiation. Examining trends in laboratory values over time could identify early signals of non-adherence and present an opportunity for clinicians to intervene. In conclusion, prolonged biochemical control was achieved by continued cinacalcet therapy and factors associated with discontinuation and reinitiation indicate persistent use of and adherence to cinacalcet are impacted by a range of both modifiable and immutable factors.

## Additional file

Additional file 1: Figure S1. Consort construction. **Figure S2**: Study design for identifying cinacalcet discontinuation. **Figure S3**: Study design for identifying cinacalcet reinitiation. **Table S1:** Baseline covariates. **Table S2**: Time-varying covariates. **Table S3**: Baseline characteristics overall and stratified by race and gender. **Table S4**. Time-dependent covariates by follow-up months. **Table S5**: Predictors of discontinuation and reinitiation. **Table S6**: Predictors of reinitiation sensitivity analysis. **Table S7**: Time-dependent cinacalcet dose by follow-up months. **Figure S4**: Vitamin D trends following cinacalcet initiation and discontinuation. (DOCX 377 kb)

## Abbreviations

SHPT: Secondary hyperparathyroidism
CKD: Chronic kidney disease
PTH: Parathyroid hormone
CKD-MBD: Chronic kidney disease-mineral and bone disorder
KDIGO: Kidney Disease: Improving Global Outcomes
USRDS: United States Renal Data System
ESRD: End-stage renal disease
SD: Standard deviation
HR: Hazard ratio
ICD-9: International Classification of Diseases Ninth Revision codes
CPT: Current Procedural Terminology codes
HCPCS: Healthcare Common Procedure Coding System codes

## References

[1] Cunningham J (2005). Management of secondary hyperparathyroidism. Ther Apher Dial.

[2] Goodman WG, Quarles LD (2008). Development and progression of secondary hyperparathyroidism in chronic kidney disease: lessons from molecular genetics. Kidney Int.

[3] Cunningham J, Locatelli F, Rodriguez M (2011). Secondary hyperparathyroidism: pathogenesis, disease progression, and therapeutic options. Clin J Am Soc Nephrol.

[4] CKDMBDWG, KDIGO (2009). KDIGO clinical practice guideline for the diagnosis, evaluation, prevention, and treatment of Chronic Kidney Disease-Mineral Bone Disorder (CKD-MBD). Kidney Int Suppl.

[5] Block GA, Martin KJ, de Francisco AL, Turner SA, Avram MM, Suranyi MG (2004). Cinacalcet for secondary hyperparathyroidism in patients receiving hemodialysis. N Engl J Med.

[6] Lindberg JS, Culleton B, Wong G, Borah MF, Clark RV, Shapiro WB (2005). Cinacalcet HCl, an oral calcimimetic agent for the treatment of secondary hyperparathyroidism in hemodialysis and peritoneal dialysis: a randomized, double-blind, multicenter study. J Am Soc Nephrol.

[7] Kilpatrick RD, Newsome BB, Zaun D, Liu J, Solid CA, Nieman K (2013). Evaluating real-world use of cinacalcet and biochemical response to therapy in US hemodialysis patients. Am J Nephrol.

[8] Smrzova J, Urbanek T (2010). Cinacalcet - clinical and laboratory effectiveness, concomitant treatment patterns and treatment cost: could we do better and how?. Kidney Blood Press Res.

[9] Urena P, Jacobson SH, Zitt E, Vervloet M, Malberti F, Ashman N (2009). Cinacalcet and achievement of the NKF/K-DOQI recommended target values for bone and mineral metabolism in real-world clinical practice--the ECHO observational study. Nephrol Dial Transplant.

[10] Gincherman Y, Moloney K, McKee C, Coyne DW (2010). Assessment of adherence to cinacalcet by prescription refill rates in hemodialysis patients. Hemodial Int.

[11] Lee A, Song X, Khan I, Belozeroff V, Goodman W, Fulcher N (2011). Association of cinacalcet adherence and costs in patients on dialysis. J Med Econ.

[12] Pruijm M, Teta D, Halabi G, Wuerzner G, Santschi V, Burnier M (2009). Improvement in secondary hyperparathyroidism due to drug adherence monitoring in dialysis patients. Clin Nephrol.

[13] Strippoli GF, Palmer S, Tong A, Elder G, Messa P, Craig JC (2006). Meta-analysis of biochemical and patient-level effects of calcimimetic therapy. Am J Kidney Dis.

[14] Ray WA (2003). Evaluating medication effects outside of clinical trials: new-user designs. Am J Epidemiol.

[15] R: A Language and Environment for Statistical Computing. In. Vienna, Austria: R Foundation for Statistical Computing; 2014.

[16] Newsome BB, Kilpatrick RD, Liu J, Zaun D, Solid CA, Nieman K (2013). Racial differences in clinical use of cinacalcet in a large population of hemodialysis patients. Am J Nephrol.

[17] Schmid H, Hartmann B, Schiffl H (2009). Adherence to prescribed oral medication in adult patients undergoing chronic hemodialysis: a critical review of the literature. Eur J Med Res.

[18] Investigators ET, Chertow GM, Block GA, Correa-Rotter R, Drueke TB, Floege J (2012). Effect of cinacalcet on cardiovascular disease in patients undergoing dialysis. N Engl J Med.

[19] Park H, Rascati KL, Lawson KA, Barner JC, Richards KM, Malone DC (2014). Adherence and persistence to prescribed medication therapy among Medicare part D beneficiaries on dialysis: comparisons of benefit type and benefit phase. J Manag Care Pharm.

[20] Chiu YW, Teitelbaum I, Misra M, de Leon EM, Adzize T, Mehrotra R (2009). Pill burden, adherence, hyperphosphatemia, and quality of life in maintenance dialysis patients. Clin J Am Soc Nephrol.

[21] Manley HJ, Garvin CG, Drayer DK, Reid GM, Bender WL, Neufeld TK (2004). Medication prescribing patterns in ambulatory haemodialysis patients: comparisons of USRDS to a large not-for-profit dialysis provider. Nephrol Dial Transplant.

[22] Li X, Sturmer T, Brookhart MA. Evidence of sample Use among New users of statins: implications for pharmacoepidemiology. Med Care. 2014. 10.1097/MLR.0000000000000174 PMC4141474 24984210

[23] Lauffenburger JC, Balasubramanian A, Farley JF, Critchlow CW, O’Malley CD, Roth MT (2013). Completeness of prescription information in US commercial claims databases. Pharmacoepidemiol Drug Saf.

[24] Brouwer ES, Napravnik S, Eron Jr JJ, Simpson Jr RJ, Brookhart MA, Stalzer B, et al. Validation of medicaid claims-based diagnosis of myocardial infarction using an HIV clinical cohort. Med Care. 2013. 10.1097/MLR.0b013e318287d6fd PMC4022708 23604043

