# Supplementary material for: Dynamics of cinacalcet use and biochemical control in hemodialysis patients: a retrospective New-user cohort design
Source: BMC Nephrol. 2015 Oct 29;16:175. doi: 10.1186/s12882-015-0174-6 (PMC4625889; doi:10.1186/s12882-015-0174-6)
Supplement: Additional file 1: Figure S1. — Consort construction. Figure S2: Study design for identifying cinacalcet discontinuation. Figure S3: Study design for identifying cinacalcet reinitiation. Table S1: Baseline covariates. Table S2: Time-varying covariates. Table S3: Baseline characteristics overall and stratified by race and gender. Table S4. Time-dependent covariates by follow-up months. Table S5: Predictors of discontinuation and reinitiation. Table S6: Predictors of reinitiation sensitivity analysis. Table S7: Time-dependent cinacalcet dose by follow-up months. Figure S4: Vitamin D trends following cinacalcet initiation and discontinuation. (DOCX 377 kb) [file 12882_2015_174_MOESM1_ESM.docx]

**Supplemental Material**

Figure S1. Consort construction

Figure S2. Study design for identifying cinacalcet discontinuation

Figure S3. Study design for identifying cinacalcet reinitiation

Table S1. Baseline covariates

Table S2. Time-varying covariates

Table S3. Baseline characteristics overall and stratified by race and gender

Table S4. Time-dependent covariates by follow-up months

Table S5. Predictors of discontinuation and reinitiation

Table S6: Predictors of reinitiation sensitivity analysis

Table S7: Time-dependent cinacalcet dose by follow-up months

Figure S4. Vitamin D trends following cinacalcet initiation and discontinuation

**Figure S1. Cohort construction**

Final eligible subjects

(N = 17,763)

Center-based, hemodialysis patients with Medicare coverage who initiated cinacalcet between 01/01/2007 and 12/31/2010

(N = 62,275)

Exclusions:

- Age < 18 years (N = 1,411)
- Dialysis vintage < 9 months (N = 7,207)
- In the baseline period did not receive in-center hemodialysis (N = 5,972)
- In the baseline period did not have continuous enrollment in Medicare Parts A, B, and D (N = 11,557)
- Received a parathyroidectomy in the baseline period or 1^st^ month of exposure (N = 59)
- Moved to another dialysis facility before follow-up (N = 215)
- Insufficient amount of claims during baseline (N = 744)
- Less than 9 dialysis sessions in the last month of baseline = (N = 13,569)
- Missing baseline lab values = (N = 47)
- Received cinacalcet in the prior 6 months (N = 3,731)

**Figure S2. Study design for identifying cinacalcet discontinuation**

**Figure S3. Study design for identifying cinacalcet reinitiation**

**Time varying covariate assessment**

**Baseline covariate assessment**

Discontinued; start follow-up

Cinacalcet reinitiated in period 4.

Use baseline and time varying covariates assessed in periods 3 to estimate the effect of covariates and laboratory values on risk of cinacalcet reinitiation during period 4.

Reinitiated

**Period 4**

30 days

**Period 3**

30 days

**Period 2**

30 days

**Period 1**

30 days

**30-days**

**6 months**

**New user 1^st^ fill**

**Table S1. Baseline covariates**

We evaluated the following covariates at baseline (6 months prior to new cinacalcet use). For the comorbid conditions identified using information provided from the Medicare claims data (part A or B), we considered comorbid conditions as present if at least one inpatient, home health, or skilled nursing facility claim, or at least two outpatient or physician/supplier claims separated by at least 7 days are identified during the baseline period.

| **Type of Variable** | **Measurement and other notes** |
| --- | --- |
| **Demographics** | |
| Patient age | Included in model as a categorical variable with 5-year age groups |
| Patient sex | Indicator for female sex |
| Patient race | As reported on the Medical Evidence form (CMS-2728)  Categories: Black, Non-Black, Hispanic, Non-Hispanic |
| Dialysis vintage | Time since start of renal replacement therapy  Categories: < 1 year, <1-3 years, <3-5 years, > 5 years |
| Cause of end-stage renal disease | Classify as diabetes, hypertension, glomerulonephritis, or other |
| Body mass index | As reported in the clinical data or on the Medical Evidence form (CMS-2728) |
| Medicaid eligibility | As reported on the Medical Evidence form (CMS-2728) |
| Low income subsidy | Reported as a categorical variable for cost share for each enrollment month. Categories include:  Category 1 = LIS, 100% premium-subsidy and high copayment  Category 2 = LIS, 100% premium-subsidy and 15% copayment  Category 3 = LIS, 75% premium-subsidy and 15% copayment  Category 4 = LIS, 50% premium-subsidy and 15% copayment  Category 5 = LIS, 25% premium-subsidy and 15% copayment  Category 6 = LIS, 75% premium-subsidy and 15% copayment  Category 7 = LIS, 50% premium-subsidy and 15% copayment  Category 8 = LIS, 25% premium-subsidy and 15% copayment |
| **Laboratory Values** | |
| Serum Parathyroid Hormone (pg/mL) | Most proximal serum parathyroid hormone value from baseline to index date  Categories:  Low <150 pg/mL  Normal 150-300 pg/mL  High >300 pg/mL |
| Corrected serum calcium level (mg/dL) | Most proximal serum calcium value from baseline to index date  Categories:  Low <7.5 mg/dL  Normal 7.6-9.5 mg/dL  High >9.5 mg/dL |
| Serum phosphorus level (mg/dL) | Most proximal serum phosphorus value from baseline to index date  Categories:  Low <3.5 mg/dL  Normal 3.5-5.5 mg/dL  High 5.5 mg/dL |
| Serum Albumin (g/dL) | Most proximal serum albumin value from baseline to index date  Categories:  Low <3.2 g/dL  Normal > 3.2 g/dL |
| **Comorbidities** |  |
| Rheumatoid arthritis / collagen vascular disease | ICD-9  701.0, 710.xx, 714.xx, 720.xx, 725.xx |
| Diabetes | ICD-9  250.xx |
| Angina | ICD-9  413.x |
| Coronary artery disease / Atherosclerosis | ICD-9  414.0x, 429.2x, 429.5x, 429.7x, 440.x |
| Cerebrovascular disease | ICD-9  342, 344.81, 430-438, 997.02, V12.54 |
| Myocardial infarction | ICD-9  410.x, 411.x |
| Malignancy | ICD-9  140.xx – 172.xx, 179.xx – 199.xx, 174.0 – 175.9x, 202.0 – 202.3x, 202.50 – 203.01, 200.xx, 201.xx, V10, 173.3x,173.9x, 232.9x, 233.0, 233.1x, 338.3x, 799.4x, 203.8x, 238.6x, 273.3x, V67.2x, 789.51, 795.82 |
| Congestive heart failure | ICD-9  398.91, 402.01, 402.11, 402.91, 404.01, 404.03, 404.11, 404.13, 404.91, 404.93, 428, 785.51, 425.4x-425.9x  HCPCS  G8027, G8028 |
| Chronic obstructive pulmonary disease and asthma | ICD-9  491.x, 492.x, 493.x, 494.x, 496.x, 510.x |
| Fracture | ICD-9  805.xx-828.xx |
| Gastrointestinal bleed | ICD-9  578.xx |
| Hyperlipidemia | ICD-9  272.xx |
| Hypertension | ICD-9  401 - 405, but not in (402.11, 402.91, 404.11, 404.13, 404.91, 404.93) |
| Liver disease | ICD-9  070.32, 070.33, 070.54, 456.20, 456.21, 456.0, 456.1x, 571.0, 571.2x - 571.6x, 571.8x, 571.9x, 572.3x, 572.8x, V42.7, 570, 573.1-573.3 |
| Hyperthyroidism | ICD-9  242.xx |
| Peripheral vascular disease | ICD-9  440.2x, 440.3x, 440.8x, 440.9x, 443.9x |
| Peptic ulcer disease | ICD-9  530.2, V12.71, 531 – 534 |
| Parathyroidectomy | ICD-9 hospital procedure codes  06.81, 06.89 |
| **Concomitant Medications** |  |
| Number of concomitant medications at time of new cinacalcet use | Categories  1-5  6-10  >11 |
| Intravenous vitamin D | Categories  Yes  No  Vitamin D intravenous:  J0635 (calcitriol 1mcg), J0636 (calcitriol 0.1mcg), J2500 (Paricalcitol 5mcg), J2501 (Paricalcitol 1mcg), J1270 (Doxercalciferol 1mcg) |
| Oral phosphate binder use | Categorical variable:  Present yes or no  Sevelamer hydrochloride (Renagel®)  Sevelamer carbonate (Renvela®)  Lanthanum carbonate (Fosrenol®)  Calcium acetate (PhosLo®) |

**Table S2: Time-varying covariates**

We evaluated the following time-varying covariates at 30-day intervals following the start of follow-up. Specific information concerning identification of comorbid conditions identified using information provided from the Medicare claims data (part A or B) is provided in the table below.

| **Type of Variable** | **Measurement and other notes** |
| --- | --- |
| **Laboratory Values** | |
| Serum Parathyroid Hormone (pg/mL) | The serum parathyroid hormone value during the time-varying covariate assessment period that is most proximal to the estimated discontinuation or reinitiation date. |
| Corrected serum calcium level (mg/dL) | The serum calcium value during the time-varying covariate assessment period that is most proximal to the estimated discontinuation or reinitiation date. |
| Serum phosphorus level (mg/dL) | The serum phosphorus value during the time-varying covariate assessment period that is most proximal to the estimated discontinuation or reinitiation date. |
| Serum Albumin (g/dL) | The serum albumin value during the time-varying covariate assessment period that is most proximal to the estimated discontinuation or reinitiation date. |
| **Concomitant Medications** | |
| Intravenous Vitamin D use | Intravenous Paricalcitol and doxercalciferol doses were converted to intravenous calcitriol-equivalent doses according to the following conversion ratios:  4.6: 1 for paricalcitol: calcitriol and 3.1: 1 for doxercalciferol: calcitriol |
| Oral phosphate binder use | Categorical variable  Present yes or no  Sevelamer hydrochloride (Renagel®)  Sevelamer carbonate (Renvela®)  Lanthanum carbonate (Fosrenol®)  Calcium acetate (PhosLo®) |
| **Cardiovascular Events** | |
| Acute myocardial infarction | ICD-9  410.xx in any diagnosis field of an inpatient claim |
| Congestive heart failure | ICD-9  428.xx in any diagnosis field of an inpatient claim |
| Stroke | ICD-9 codes 430, 431, 433.x1, 434, and 436 in any diagnosis field of an inpatient claim |
| Peripheral vascular disease event | ICD-9 and CPT codes in any diagnosis field of Part A or B files  Lower extremity amputation  84.1×, 84.91  27295, 27590-92, 27598, 27880-82, 27888-89, 28800, 28805  Lower extremity peripheral angioplasty, atherectomy, or endarterectomy  38.18, 39.50  35302-06,35331, 35351, 35355, 35361, 35363, 35371-72, 35381, 35452, 35454, 35456, 35459, 35470, 35472-74, 35481-35483, 35485, 35491-35493, 35495  Lower extremity peripheral bypass  39.25, 39.29  35521, 35533, 35537-41, 35546, 35548-49, 35551, 35556, 35558, 35563, 35565-66, 35571, 35582-83, 35585, 35587, 35621, 35623, 35637-38, 35641,35646-47, 35651, 35654, 35656, 35661, 35663, 35665-66, 35671  Repair, exploration, revision, resection of lower extremity arteries, or thrombectomy of graft  38.08, 38.38, 38.48, 39.49, 39.56, 39.57, 39.58  35226, 35256, 35286,35700, 35721, 35741, 35876, 35879, 35881, 35883, 35884  Non-coronary vessel percutaneous transluminal mechanical thrombectomy, or stents  39.90  37184-86, 37205-08 |
| Cardiovascular mortality | Primary causes of death on CMS-Form 2746 coded as  23 (acute myocardial infarction)  25 (pericarditis, including cardiac tamponade)  26 (atherosclerotic heart disease)  27 (cardiomyopathy)  28 (cardiac arrhythmia)  29 (cardiac arrest, cause unknown)  30 (valvular heart disease)  31 (pulmonary edema due to exogenous fluid)  32 (congestive heart failure)  36 (cerebrovascular accident including intracranial hemorrhage) |
| **Other Events** | |
| Parathyroidectomy | ICD-9 hospital procedure codes  06.81, 06.89 in any diagnosis field of an inpatient claim |
| All cause mortality |  |
| **Adverse Events** | |
| Nausea, Vomiting, diarrhea | ICD-9  564.5, 787.91, 787.0x  Considered present if at least one inpatient, home health, or skilled nursing facility claim, or at least two outpatient or physician/supplier claims separated by at least 7 days are identified during the 30-day look back period.  Categorical variable; yes or no |
| Seizure | ICD-9  333.2, 345, 345.0. 345.00, 345.01, 345.1, 345.10, 345.11, 345.2, 345.3, 345.4, 345.40, 345.41, 345.5, 345.50, 345.51, 345.8, 345.80, 345.81, 345.90, 345.91, 780.3  Considered present if at least one inpatient, home health, or skilled nursing facility claim, or at least two outpatient or physician/supplier claims separated by at least 7 days are identified during the 30-day look back period.  Categorical variable; yes or no |
| Hypocalcemia | Corrected serum calcium below 7.5mg/dl  The serum calcium value during the time-varying covariate assessment period that is most proximal to the estimated discontinuation or reinitiation date    Categorical variable; yes or no |
| Low parathyroid hormone level | Parathyroid hormone level below 150 pg/mL  The serum parathyroid hormone value during the time-varying covariate assessment period that is most proximal to the estimated discontinuation or reinitiation date  Categorical variable; yes or no |
| **Other Covariates** | |
| Current vascular access | Using access information obtained from the clinical data, we classified patients as having a catheter, fistula, or graft |
| Acute Care Hospitalization, in days | Determined from the USRDS file institutional claims file |
| Co-pay | Cost of cinacalcet prescription using Part D claims data |
| Donut Hole status | Determined from Medicare Part D file “Benefit Phase” variable.  Categories:  Covered prescription  Cinacalcet prescription fill resulted in entrance into the gap period  Cinacalcet prescription was filled while they were in the gap period  Cinacalcet prescription fill resulted in exiting the gap period  Cinacalcet prescription fill resulted in going through the gap period  Other |

**Table S3. Baseline characteristics overall and stratified by race and gender**

| **Characteristic^1^** | **Total** | **Female** | **Male** | **African American** |
| --- | --- | --- | --- | --- |
| **Demographics** | | | | |
| Patients, N | 17,763 | 8,764 | 8,999 | 9,555 |
| Age, mean (SD), years^2^ | 56.7 (14.5) | 59.1 (14.8) | 54.4 (13.9) | 55.5 (14.1) |
| Time on dialysis, mean (SD), years^2^ | 4.5 (4.3) | 4.4 (4.1) | 4.7 (4.4) | 4.8 (4.3) |
| Race, N (%) |  |  |  |  |
| White | 7,242 (40.8) | 3,436 (39.2) | 3,806 (42.3) | n/a |
| African American | 9,555 (53.8) | 4,856 (55.4) | 4,699 (52.2) | n/a |
| Other Race | 966 (5.4) | 472 (5.4) | 494 (5.5) | n/a |
| Cause of ESRD, N (%) |  |  |  |  |
| Diabetes | 7,629 (42.9) | 4,233 (48.3) | 3,396 (37.7) | 3,691 (38.6) |
| Hypertension | 5,612 (31.6) | 2,458 (28.0) | 3,154 (35.0) | 3,789 (39.7) |
| Glomerulonephritis | 2,236 (12.6) | 1,067 (12.2) | 1,169 (13.0) | 1,105 (11.6) |
| Other | 2,286 (12.9) | 1,006 (11.5) | 1,280 (14.2) | 970 (10.2) |
| Body Mass Index, mean (SD), kg/m^2^ | 28.0 (7.3) | 28.7 (7.8) | 27.4 (6.7) | 28.1 (7.4) |
| **Financial Considerations** | | | | |
| Medicaid, N (%) | 12,206 (68.7) | 6,351 (72.5) | 5,855 (65.1) | 6,890 (72.1) |
| Low-income subsidy, N (%) | 14,906 (83.9) | 7,515 (85.7) | 7,391 (82.1) | 8,414 (88.1) |
| Concomitant medications, N (%)^3^ | 4.7 (3.6) | 5.1 (3.7) | 4.4 (3.5) | 4.4 (3.5) |
| **Biochemical Values** | | | | |
| Albumin, mean (SD), g/dL^4^ | 3.9 (0.4) | 3.8 (0.4) | 4.0 (0.4) | 3.9 (0.4) |
| Calcium, mean (SD), mg/dL^4^ | 9.4 (0.7) | 9.4 (0.7) | 9.4 (0.7) | 9.4 (0.7) |
| Phosphorus, mean (SD), mg/dL^4^ | 5.9 (1.7) | 5.8 (1.7) | 6.0 (1.7) | 5.8 (1.7) |
| Parathyroid hormone, mean (SD), pg/mL^4^ | 642 (519) | 640 (519) | 644 (520) | 687 (568) |
| **Comorbidities** | | | | |
| Angina, N (%) | 349 (2.0) | 185 (2.1) | 164 (1.8) | 180 (1.9) |
| Congestive heart failure, N (%) | 4,823 (27.2) | 2,592 (29.6) | 2,231 (24.8) | 2,611 (27.3) |
| Coronary artery disease / atherosclerosis, N (%) | 4,703 (26.5) | 2,434 (27.8) | 2,269 (25.2) | 2,250 (23.5) |
| Cerebrovascular disease, N (%) | 1,891 (10.6) | 1,087 (12.4) | 804 (8.9) | 1,070 (11.2) |
| Myocardial infarction, N (%) | 679 (3.8) | 363 (4.1) | 316 (3.5) | 362 (3.8) |
| Hypertension, N (%) | 12,393 (69.8) | 6,481 (74.0) | 5,912 (65.7) | 6,909 (72.3) |
| Peripheral vascular disease, N (%) | 2,352 (13.2) | 1,208 (13.8) | 1,144 (12.7) | 1,217 (12.7) |
| Hyperlipidemia, N (%) | 4,658 (26.2) | 2,519 (28.7) | 2,139 (23.8) | 2,292 (24.0) |
| Chronic obstructive pulmonary disease and asthma, N (%) | 2,727 (15.4) | 1,530 (17.5) | 1,197 (13.3) | 1,350 (14.1) |
| Rheumatoid arthritis / collagen vascular disease, N (%) | 511 (2.9) | 398 (4.5) | 113 (1.3) | 319 (3.3) |
| Diabetes, N (%) | 10,052 (56.6) | 5,491 (62.7) | 4,561 (50.7) | 5,121 (53.6) |
| Fracture, N (%) | 697 (3.9) | 451 (5.1) | 246 (2.7) | 276 (2.9) |
| Gastrointestinal bleed, N (%) | 504 (2.8) | 285 (3.3) | 219 (2.4) | 303 (3.2) |
| Hyperthyroidism, N (%) | 105 (0.59) | 73 (0.83) | 32 (0.36) | 51 (0.53) |
| Peptic ulcer disease, N (%) | 285 (1.6) | 149 (1.7) | 136 (1.5) | 150 (1.6) |
| Liver disease, N (%) | 640 (3.6) | 257 (2.9) | 383 (4.3) | 352 (3.7) |
| Cancer, N (%) | 908 (5.1) | 424 (4.8) | 484 (5.4) | 439 (4.6) |
| **Dialysis Care** | | | | |
| Phosphorus binder drug, N (%)^5^ | 14,135 (79.6) | 7,037 (80.3) | 7,098 (78.9) | 7,383 (77.3) |
| Catheter access, N (%) | 3,351 (18.9) | 1,994 (22.8) | 1,357 (15.1) | 1,793 (18.8) |
| Mean intravenous vitamin D dosage, micrograms (SD)^6^ | 12.5 (10.3) | 11.9 (9.9) | 13.1 (10.7) | 14.6 (11.2) |

*Note:* Conversion factors for units: Calcium in mg/dL to mmol/L, x0.2495; phosphorus in mg/dL to mmol/L, x0.3229.

^1^Characteristics were identified using information from Medicare Part A or B claims. A characteristic was considered present if at least one inpatient, home health, or skilled nursing facility claim, or at least two outpatient or physician/ supplier claims separated by at least 7 days, were identified during the 6-month baseline period.

^2^Age and time on dialysis are at the time of cinacalcet initiation.

^3^Concomitant medications are the mean number of concomitant medications at the time of cinacalcet initiation.

^4^Laboratory values were those most proximal to the index date during the baseline period.

^5^Phosphate binders included in the analysis: Sevelamer hydrochloride, sevelamer carbonate, lanthanum carbonate, and calcium acetate.

^6^Mean intravenous vitamin D dose per person in the last month of the baseline period. Paricalcitol and doxercalciferol doses were converted to calcitriol-equivalent doses according to the following conversion ratios: 4.6: 1 for paricalcitol: calcitriol and 3.1: 1 for doxercalciferol: calcitriol.

Table S4. Time-dependent covariates by follow-up months

|  | **Total** | **Year 1** | | | | **Year 2** | | **Year 3** | **Year 4** |
| --- | --- | --- | --- | --- | --- | --- | --- | --- | --- |
| **Characteristics** | **48 months** | **Month 1** | **Month 2** | **Month 3** | **Month 7** | **Month 1** | **Month 7** | **Month 1** | **Month 1** |
| Number of intervals, N | 111,047 | 17,763 | 11,436 | 9,292 | 5,210 | 2,649 | 1,491 | 900 | 262 |
| Discontinuation, N (%) | 12,521 (11.3) | 5,944 (33.5) | 1,736 (15.2) | 927 (10.0) | 345 (6.6) | 143 (5.4) | 33 (2.2) | 30 (3.3) | 5 (1.9) |
| Average number of days in an interval | 26.8 | 20.4 | 26.5 | 27.6 | 28.0 | 28.4 | 28.9 | 28.6 | 28.5 |
| Albumin in the follow-up period, mean (SD), median, g/dL | 3.9 (0.4),  3.9 | 3.9 (0.4),  3.9 | 3.9 (0.4),  3.9 | 3.9 (0.4),  3.9 | 3.9 (0.4), 3.9 | 3.9 (0.4), 4.0 | 3.9 (0.4), 4.0 | 3.9 (0.4), 4.0 | 4.0 (0.4), 4.0 |
| Calcium in the follow-up period, mean (SD), median, mg/dL | 8.9 (0.7),  8.9 | 9.0 (0.7),  9.0 | 8.9 (0.8),  8.9 | 8.9 (0.7),  8.9 | 8.9 (0.7), 8.9 | 8.9 (0.7), 8.9 | 8.9 (0.7), 8.9 | 8.8 (0.6), 8.9 | 8.9 (0.7), 8.8 |
| Phosphorus in the follow-up period, mean (SD), median, mg/dL | 5.3 (1.6),  5.1 | 5.6 (1.7),  5.4 | 5.5 (1.7),  5.2 | 5.4 (1.7),  5.2 | 5.3 (1.6), 5.1 | 5.2 (1.6), 5.0 | 5.1 (1.6), 4.9 | 5.1 (1.6), 4.9 | 5.0 (1.5), 4.8 |
| PTH in the follow-up period, mean (SD), median, pg/mL | 415 (428), 291 | 497 (513), 353 | 450 (469), 313 | 424 (436), 292 | 392 (389), 280 | 396 (398), 279 | 364 (395), 264 | 367 (414), 265 | 352 (282), 279 |
| Congestive heart failure,^1^ N (%) | 4,273 (3.8) | 717 (4.0) | 551 (4.8) | 397 (4.3) | 209 (4.0) | 85 (3.2) | 52 (3.5) | 29 (3.2) | 9 (3.4) |
| MI, ^1^ N (%) | 766 (0.69) | 127 (0.71) | 107 (0.94) | 80 (0.86) | 29 (0.56) | 12 (0.45) | 14 (0.94) | 4 (0.44) | 1 (0.38) |
| Stroke, ^1^ N (%) | 799 (0.72) | 99 (0.56) | 107 (0.94) | 76 (0.82) | 52 (1.00) | 10 (0.38) | 8 (0.54) | 8 (0.89) | 1 (0.38) |
| Peripheral vascular disease, ^2^ N (%) | 2,148 (1.9) | 279 (1.6) | 263 (2.3) | 193 (2.1) | 97 (1.9) | 43 (1.6) | 31 (2.1) | 14 (1.6) | 4 (1.5) |
| Phosphorus binder drug in the follow-up period, N (%) | 62,616 (56.4) | 9,278 (52.2) | 6,127 (53.6) | 4,996 (53.8) | 2,942 (56.5) | 1,552 (58.6) | 911 (61.1) | 547 (60.8) | 143 (54.6) |
| Recent catheter access in the follow-up period, N (%) | 16,716 (15.2) | 3,186 (18.0) | 2,015 (17.8) | 1,540 (16.8) | 747 (14.6) | 359 (13.7) | 176 (12.0) | 98 (11.0) | 29 (11.2) |
| Intravenous vitamin D dosage in the last 30 days, mean (SD), median, micrograms ^4^ | 12.0 (10.8), 9.9 | 12.8 (10.4), 11.1 | 12.2 (10.5), 10.1 | 12.0 (10.5), 9.9 | 11.5 (10.7), 9.1 | 11.6 (10.8), 9.4 | 12.3 (11.6), 10.0 | 12.7 (11.9), 9.9 | 12.0 (12.3), 9.8 |
| Days in hospital, mean (SD), median | 1.0 (3.5),  0.0 | 0.78 (2.69), 0.0 | 1.2 (3.6),  0.0 | 1.2 (4.0),  0.0 | 1.1 (3.8), 0.0 | 0.94 (3.33), 0.0 | 0.81 (2.88), 0.0 | 0.89 (3.50), 0.0 | 1.1 (3.9), 0.0 |
| Nausea, Vomiting, diarrhea, ^5^ N (%) | 1,308 (1.2) | 190 (1.1) | 156 (1.4) | 134 (1.4) | 60 (1.2) | 18 (0.68) | 12 (0.80) | 10 (1.1) | 3 (1.1) |
| Seizure, ^5^ N (%) | 765 (0.69) | 89 (0.50) | 101 (0.88) | 66 (0.71) | 35 (0.67) | 21 (0.79) | 8 (0.54) | 3 (0.33) | 0 |
| Monthly copay, mean (SD), median, dollars | 22.9 (93.3),  3.1 | 29.3 (103.6),  3.2 | 29.1 (108.6),  3.1 | 29.9 (111.2), 3.1 | 22.1 (88.6), 3.1 | 15.7 (71.5), 3.0 | 14.2 (69.0), 0.0 | 12.4 (67.0), 1.7 | 13.4 (66.5), 0.0 |
| Concomitant number of medications in past month, mean (SD), median | 5.8 (3.7),  5.0 | 5.3 (3.6),  5.0 | 5.6 (3.6),  5.0 | 5.7 (3.7),  5.0 | 6.0 (3.7), 5.0 | 6.0 (3.8), 6.0 | 6.1 (3.9), 6.0 | 6.2 (4.1), 5.5 | 6.0 (3.8), 5.5 |
| Hypocalcemia (<7.5 mg/dL), N (%) | 1,934 (1.8) | 331 (1.9) | 244 (2.1) | 167 (1.8) | 94 (1.8) | 49 (1.9) | 16 (1.1) | 16 (1.8) | 4 (1.5) |
| Low PTH (<150 pg/mL), N (%) | 17,980 (16.7) | 2,514 (16.2) | 1,946 (17.5) | 1,646 (17.8) | 844 (16.4) | 420 (16.1) | 261 (17.7) | 125 (14.0) | 36 (13.9) |
| Gap period activity, ^5^ N (%) | 44,708 (41.3) | 7,375 (41.5) | 5,594 (50.0) | 4,736 (52.8) | 1,996 (39.7) | 930 (36.5) | 432 (29.6) | 322 (36.8) | 97 (38.2) |

*Note:* Conversion factors for units: Calcium in mg/dL to mmol/L, x0.2495; phosphorus in mg/dL to mmol/L, x0.3229.

^1^Myocardial infarction, congestive heart failure, stroke, and parathyroidectomy were considered in any diagnosis field of an inpatient claim only.

^2^Peripheral vascular disease was considered in any diagnosis field of Medicare Part A and B files.

^3^Phosphate binders included in the analysis: Sevelamer hydrochloride, sevelamer carbonate, lanthanum carbonate, and calcium acetate

^4^Paricalcitol and doxercalciferol doses were converted to calcitriol-equivalent doses according to the following conversion ratios: 4.6: 1 for paricalcitol: calcitriol and 3.1: 1 for doxercalciferol: calcitriol.

^5^Seizure, nausea, vomiting, and diarrhea considered present if at least one inpatient, home health, or skilled nursing facility claim, or at least two outpatient or physician/supplier claims separated by at least 7 days are identified during the 30-day look back period.

^6^Gap period activity was defined as the percentage of participants who in the prior 30-day period had a prescription filled that resulted in entering, being in, exiting, or going through the doughnut hole.

**Table S5. Predictors of discontinuation and reinitiation**

| Characteristic^1^ | Discontinuation (HR, 95% CI) | Reinitiation (HR, 95% CI) |
| --- | --- | --- |
| Number of time intervals for analysis, N (%) | 100,706 (90.7%) | 78,789 (96.0%) |
| **Demographics** | | |
| Age, years, reference 46-55 |  |  |
| <45 | 0.92 (0.85, 1.00) | 0.95 (0.90, 1.02) |
| 56-65 | 1.05 (0.97, 1.13) | 0.98 (0.92, 1.04) |
| 66-75 | 1.05 (0.97, 1.15) | 0.90 (0.85, 0.97) |
| >75 | 0.98 (0.88, 1.09) | 0.95 (0.87, 1.04) |
| Time on dialysis, years, reference <1 |  |  |
| 1-3 | 1.15 (1.01, 1.30) | 1.00 (0.91, 1.11) |
| >4 | 1.14 (1.00, 1.30) | 1.03 (0.92, 1.14) |
| Female | 1.07 (1.01, 1.13) | 1.00 (0.96, 1.05) |
| African American | 1.04 (0.98, 1.10) | 1.08 (1.03, 1.13) |
| Cause of ESRD, reference diabetes mellitus |  |  |
| Hypertension | 1.02 (0.94, 1.10) | 1.04 (0.98, 1.11) |
| Glomerulonephritis | 1.01 (0.90, 1.12) | 1.08 (0.99, 1.17) |
| Other | 0.96 (0.87, 1.06) | 1.03 (0.95, 1.12) |
| Body mass index, kg/m^2^, reference normal |  |  |
| Underweight | 1.07 (0.93, 1.23) | 0.98 (0.87, 1.10) |
| Overweight | 1.03 (0.96, 1.10) | 1.03 (0.98, 1.09) |
| Obese | 0.98 (0.91, 1.04) | 1.11 (1.05, 1.17) |
| **Financial considerations** | | |
| Medicaid | 1.03 (0.96, 1.11) | 0.96 (0.91, 1.02) |
| Low-income subsidy | 0.76 (0.68, 0.85) | 1.32 (1.22, 1.43) |
| Concomitant medications in baseline period^2^ | 0.98 (0.97, 0.99) | 1.00 (0.99, 1.01) |
| Concomitant medications in follow-up period^2^ | 0.96 (0.95, 0.97) | 0.98 (0.97, 0.99) |
| Copay in follow-up period^3^ | 1.04 (1.01, 1.06) | 1.04 (1.02, 1.06) |
| Last benefit phase in follow-up, reference: covered^4^ |  |  |
| Entering the gap period | 1.19 (1.00, 1.41) | 1.01 (0.85, 1.21) |
| Exiting or going through gap  period | 0.98 (0.77, 1.23) | 1.03 (0.81, 1.32) |
| In the gap period | 1.10 (1.04, 1.16) | 1.01 (0.96, 1.06) |
| **Biochemical values** | | |
| Albumin in baseline period, reference: <3.3 g/dL |  |  |
| 3.3-3.9 g/dL | 1.11 (0.97, 1.28) | 1.13 (1.00, 1.27) |
| >3.9 g/dL | 1.05 (0.91, 1.21) | 1.09 (0.96, 1.23) |
| Albumin in follow-up period, reference: <3.3 g/dL |  |  |
| 3.3-3.9 g/dL | 0.85 (0.76, 0.95) | 1.13 (1.02, 1.25) |
| >3.9 g/dL | 0.78 (0.69, 0.88) | 1.23 (1.10, 1.36) |
| Phosphorus in baseline period, mg/dL | 1.02 (1.00, 1.04) | 0.98 (0.96, 0.99) |
| Phosphorus in follow-up period, mg/dL | 1.02 (1.00, 1.04) | 0.99 (0.98, 1.01) |
| Parathyroid hormone in baseline period, pg/mL^5^ | 1.00 (0.99, 1.01) | 1.00 (0.99, 1.00) |
| Parathyroid hormone in follow-up period, reference: >600 pg/mL |  |  |
| <150 pg/mL | 1.24 (1.12, 1.37) | 0.70 (0.64, 0.76) |
| 150-300 pg/mL | 0.91 (0.83, 0.99) | 0.71 (0.66, 0.75) |
| 301-600 pg/mL | 0.89 (0.82, 0.97) | 0.85 (0.80, 0.90) |
| Parathyroid hormone in follow-up period, change in quintiles, reference: no change^6^ |  |  |
| Increase | 1.15 (1.07, 1.23) | 1.08 (1.03, 1.14) |
| Decrease | 0.90 (0.84, 0.97) | 1.12 (1.06, 1.19) |
| Calcium in baseline period, mg/dL | 0.95 (0.91, 0.99) | 1.16 (1.12, 1.20) |
| Calcium in follow-up period, reference: >8.7mg/dL^7^ |  |  |
| <7.5mg/dL | 1.07 (0.89, 1.29) | 1.12 (0.91, 1.39) |
| 7.5-8.7mg/dL | 0.85 (0.80, 0.91) | 1.26 (1.19, 1.33) |
| Calcium in follow-up period, change in quintiles, reference: no change^6^ |  |  |
| Increase | 1.24 (1.16, 1.32) | 1.07 (1.02, 1.13) |
| Decrease | 0.94 (0.88, 1.00) | 1.04 (0.99, 1.10) |
| **Comorbidities** | | |
| Angina in baseline period | 1.27 (1.07, 1.52) | 1.06 (0.91, 1.23) |
| Congestive heart failure in baseline period | 1.06 (0.99, 1.13) | 0.96 (0.91, 1.01) |
| Congestive heart failure in follow-up period | 1.01 (0.90, 1.14) | 1.11 (0.94, 1.31) |
| Coronary artery disease / atherosclerosis in baseline period | 1.01 (0.95, 1.09) | 0.93 (0.88, 0.99) |
| Cerebrovascular disease in baseline period | 0.94 (0.86, 1.02) | 1.00 (0.93, 1.07) |
| Myocardial infarction in baseline period | 1.00 (0.87, 1.15) | 1.02 (0.90, 1.15) |
| Myocardial infarction in follow-up period | 1.05 (0.83, 1.32) | 0.65 (0.42, 0.99) |
| Stroke in follow-up period | 1.30 (1.05, 1.60) | 0.82 (0.55, 1.20) |
| Hypertension in baseline period | 1.12 (1.05, 1.19) | 1.03 (0.98, 1.08) |
| Peripheral vascular disease in baseline period | 1.02 (0.94, 1.11) | 1.11 (1.03, 1.19) |
| Peripheral vascular disease in follow-up period | 0.99 (0.85, 1.16) | 0.91 (0.76, 1.09) |
| Hyperlipidemia in baseline period | 1.01 (0.95, 1.07) | 1.06 (1.01, 1.12) |
| Chronic obstructive pulmonary disease and asthma in baseline period | 1.00 (0.93, 1.08) | 1.00 (0.94, 1.06) |
| Rheumatoid arthritis in baseline period | 1.15 (0.98, 1.35) | 1.05 (0.93, 1.19) |
| Diabetes in baseline period | 1.05 (0.98, 1.14) | 0.98 (0.92, 1.04) |
| Fracture in baseline period | 1.01 (0.88, 1.15) | 1.03 (0.92, 1.15) |
| Gastrointestinal bleed in baseline period | 0.81 (0.69, 0.96) | 0.96 (0.84, 1.11) |
| Hyperthyroidism in baseline period | 0.96 (0.70, 1.33) | 1.02 (0.76, 1.38) |
| Peptic ulcer disease in baseline period | 1.21 (0.99, 1.48) | 0.91 (0.77, 1.09) |
| Liver disease in baseline period | 1.07 (0.93, 1.22) | 1.07 (0.96, 1.20) |
| Cancer in baseline period | 0.96 (0.85, 1.08) | 1.02 (0.92, 1.13) |
| Nausea, vomiting, diarrhea in follow-up period | 1.09 (0.91, 1.32) | 1.05 (0.85, 1.31) |
| Seizure in follow-up period | 1.18 (0.93, 1.49) | 1.11 (0.82, 1.50) |
| **Dialysis care** | | |
| Intravenous vitamin D in baseline period ^8^ | 1.01 (0.98, 1.05) | 0.98 (0.96, 1.00) |
| Intravenous vitamin D in follow-up period ^8^ | 0.94 (0.91, 0.97) | 1.02 (0.99, 1.04) |
| Phosphorus binder drug in baseline period^9^ | 1.02 (0.95, 1.10) | 1.12 (1.06, 1.18) |
| Phosphorus binder drug in follow-up period^9^ | 0.77 (0.73, 0.82) | 1.03 (0.98, 1.08) |
| Catheter access in baseline period | 0.97 (0.89, 1.07) | 1.07 (0.99, 1.15) |
| Catheter access in follow-up period | 1.10 (1.00, 1.21) | 0.83 (0.77, 0.90) |
| Most recent dose of cinacalcet, reference: 30 mg |  |  |
| 60 mg | 1.07 (1.00, 1.15) | n/a |
| 90 mg | 1.15 (1.03, 1.29) | n/a |
| Days in the hospital in follow-up period, reference: 0 days |  |  |
| 1-4 days | 2.02 (1.84, 2.22) | 0.85 (0.75, 0.96) |
| >5 days | 1.90 (1.73, 2.08) | 0.79 (0.69, 0.89) |

*Note:* Conversion factors for units: Calcium in mg/dL to mmol/L, x0.2495; phosphorus in mg/dL to mmol/L, x0.3229.

^1^Baseline characteristics were identified using information from Medicare Part A or B claims. A characteristic was considered present if at least one inpatient, home health, or skilled nursing facility claim, or at least two outpatient or physician/ supplier claims separated by at least 7 days, were identified during the 6-month baseline period. Additional information concerning baseline characteristics can be found in Table S1. Time-varying (follow-up) characteristics were evaluated at 30-day intervals following the start of follow-up. Additional information concerning time-varying (follow-up) characteristics can be found in Table S2.

^2^Concomitant medications are the number of concomitant medications at the time of cinacalcet discontinuation or reinitiation.

^3^Changes in co-pay were based on increments of $100. The last co-pay prior to discontinuation was used to predict cinacalcet reinitiation.

^4^Benefit phase reflects the status of Medicare Part D coverage at the time of the fill of cinacalcet.

^5^Changes in parathyroid hormone level were based on increments of 100 pg/mL.

^6^Distributions of parathyroid hormone and calcium were examined across all of follow-up and quintiles were based on these distributions. Increase indicates an increase to another quintile and trend of increasing laboratory levels. Decrease indicates a decrease to another quintile and a trend of decreasing laboratory levels.

^7^Results presented for prediction of reinitiation associated with follow-up calcium levels are those from the sensitivity analysis utilizing a lag time of 14 days. The calcium level recorded 14 days prior to the date of the laboratory value most proximal to discontinuation was used to predict reinitiation. All other results were not significantly changed when lag times were considered.

^8^Mean intravenous vitamin D dose was assessed in the last month of the baseline period. Changes in intravenous vitamin D dose were based in increments of 10mcg. Paricalcitol and doxercalciferol doses were converted to calcitriol-equivalent doses according to the following conversion ratios: 4.6: 1 for paricalcitol: calcitriol and 3.1: 1 for doxercalciferol: calcitriol.

^9^Phosphate binders included in the analysis: Sevelamer hydrochloride, sevelamer carbonate, lanthanum carbonate, and calcium acetate

**Table S6. Predictors of reinitiation sensitivity analysis**

A sensitivity analysis was performed to determine if reinitiation results were modified when biochemical results from 5, 7, and 14 days prior to the date of the laboratory value most proximal to reinitiation were used to predict reinitiation. This lag time could account for any delays between physician recognition of a laboratory abnormality and a decision to reinitiate cinacalcet.

| **Biochemical Parameter** | **Use of Most Proximal Value (HR, 95% CI)** | **Addition of 5 days**  **(HR, 95% CI)** | **Addition of 7 days**  **(HR, 95% CI)** | **Addition of 14 days**  **(HR, 95% CI)** |
| --- | --- | --- | --- | --- |
| Phosphorus in follow-up period, mg/dL | 0.99 (0.98, 1.01) | 0.98 (0.97, 1.00) | 0.98 (0.97, 1.00) | 0.98 (0.97, 1.00) |
| Parathyroid hormone in follow-up period, reference: >600 pg/mL |  |  |  |  |
| <150 pg/mL | 0.70 (0.64, 0.76) | 0.70 (0.65, 0.77) | 0.71 (0.65, 0.78) | 0.73 (0.67, 0.80) |
| 150-300 pg/mL | 0.71 (0.66, 0.75) | 0.71 (0.66, 0.75) | 0.72 (0.67, 0.77) | 0.73 (0.69, 0.78) |
| 301-600 pg/mL | 0.85 (0.80, 0.90) | 0.86 (0.81, 0.91) | 0.87 (0.82, 0.92) | 0.88 (0.83, 0.93) |
| Parathyroid hormone in follow-up period, change in quintiles, reference: no change^1^ |  |  |  |  |
| Increase | 1.08 (1.03, 1.14) | 1.08 (1.02, 1.13) | 1.05 (0.99, 1.10) | 1.07 (1.01, 1.13) |
| Decrease | 1.12 (1.06, 1.19) | 1.14 (1.07, 1.20) | 1.12 (1.06, 1.19) | 1.14 (1.08, 1.21) |
| Calcium in follow-up period, reference: >8.7mg/dL |  |  |  |  |
| <7.5mg/dL | 1.27 (1.03, 1.57) | 1.28 (1.04, 1.57) | 1.25 (1.02, 1.54) | 1.12 (0.91, 1.39) |
| 7.5-8.7mg/dL | 1.37 (1.30, 1.45) | 1.31 (1.23, 1.38) | 1.29 (1.22, 1.37) | 1.26 (1.19, 1.33) |
| Calcium in follow-up period, change in quintiles, reference: no change^1^ |  |  |  |  |
| Increase | 1.07 (1.02, 1.13) | 1.04 (0.99, 1.10) | 1.04 (0.99, 1.10) | 1.01 (0.95, 1.06) |
| Decrease | 1.04 (0.99, 1.10) | 1.05 (1.00, 1.11) | 1.06 (1.00, 1.11) | 1.04 (0.99, 1.09) |

*Note:* Conversion factors for units: Calcium in mg/dL to mmol/L, x0.2495; phosphorus in mg/dL to mmol/L, x0.3229.

^1^Distributions of parathyroid hormone and calcium were examined across all of follow-up and quintiles were based on these distributions. Increase indicates an increase to another quintile and trend of increasing laboratory levels. Decrease indicates a decrease to another quintile and a trend of decreasing laboratory levels.

Table S7: Time-dependent cinacalcet dose by follow-up months, N (%)

|  |  | | | | | | | | |
| --- | --- | --- | --- | --- | --- | --- | --- | --- | --- |
|  | **Total** | **Year 1** | | | | **Year 2** | | **Year 3** | **Year 4** |
| **Cinacalcet Dose** | **48 months** | **Month 1** | **Month 2** | **Month 3** | **Month 7** | **Month 1** | **Month 7** | **Month 1** | **Month 1** |
| 30 mg | 80,656 (72.6) | 14,041 (79.0) | 9,068 (79.3) | 7,235 (77.9) | 3,794 (72.8) | 1,806 (68.2) | 964 (64.7) | 566 (62.9) | 147 (56.1) |
| 60 mg | 23,093 (20.8) | 2,699 (15.2) | 1,839 (16.1) | 1,604 (17.3) | 1,098 (21.1) | 625 (23.6) | 402 (27.0) | 255 (28.3) | 87 (33.2) |
| 90 mg | 7,298 (6.6) | 1,023 (5.8) | 529 (4.6) | 453 (4.9) | 318 (6.1) | 218 (8.2) | 125 (8.4) | 79 (8.8) | 28 (10.7) |

**Figure S4. Vitamin D trends following cinacalcet initiation and discontinuation**

Paricalcitol and doxercalciferol doses were converted to calcitriol-equivalent doses according to the following conversion ratios: 4.6: 1 for paricalcitol: calcitriol and 3.1: 1 for doxercalciferol: calcitriol.

**
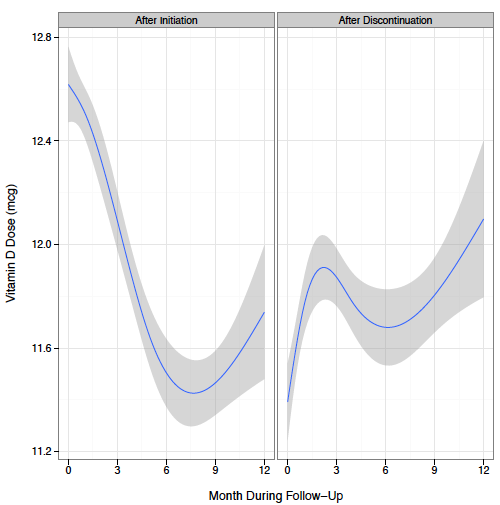
**
